# Evaluation of the Effects of Pterygium and Aging on Limbal Structure Using Optical Coherence Tomography

**DOI:** 10.3390/jcm11195879

**Published:** 2022-10-05

**Authors:** Shengwei Li, Haozhe Yu, Pu Wang, Yun Feng

**Affiliations:** 1 Department of Ophthalmology, Peking University Third Hospital, Beijing 100191, China; 2 Institute of Medical Technology, Peking University Health Science Centre, Beijing 100191, China; 3 Department of Oral and Maxillofacial Surgery, Peking University School and Hospital of Stomatology, Beijing 100081, China

**Keywords:** limbus, pterygium, aging, epithelial thickness, optical coherence tomography, OCT

## Abstract

Previous studies suggest that regions of corneal limbus may possess structural differences. We aimed to investigate the limbal changes associated with pterygium and aging via optical coherence tomography (OCT). Palisades of Vogt epithelial thickness (POV-ET) and Bowman’s membrane epithelial thickness (BM-ET) were measured at the nasal, temporal, superior, and inferior quadrants of patients with pterygium and healthy subjects of different ages. Values were expressed as a ratio that functioned as an index used to evaluate the change of limbus. Ratio values determined for quadrants of the corneal limbus were correlated highly in young healthy subjects. Further, parameter values were significantly greater than those of elder healthy subjects. In young subjects, the temporal and superior quadrants of patients with pterygium were significantly lower than those of healthy subjects. Temporal and superior quadrants of elder pterygium patients affected by both pterygium and age were significantly lower than those of healthy subjects; however, the inferior quadrant of elderly pterygium patients was significantly higher than that of age-matched healthy subjects. Our findings revealed that the thickness of limbal epithelium was negatively correlated with age, while pterygium led to the thinning of the temporal and superior limbal epithelium and inferior limbal epithelial thickening.

## 1. Introduction

Pterygium is an ocular surface disease characterized mainly by a wing-shaped growth of limbal and conjunctival tissue over the adjacent cornea. The reported prevalence of pterygium ranges from 1.2% to about 40% in different parts of the world [1]. Due to alterations in local ocular surface homeostasis, the main components of pterygium include proliferative clusters of limbal stem cells, epithelial metaplasia, active fibrovascular tissue, inflammation, and disruption of Bowman’s membrane (BM) along the invading apex of the pterygium [2]. Risk factors for pterygium include ultraviolet, age, male sex, living in rural residences, and geographical latitude [1,3]. Among these factors, ultraviolet and age are considered most important. Previous reports that revealed limbal epithelium changes throughout the pathogenesis of pterygium suggest that the condition is associated with limbal stem cell damage [4].

The limbus is an annular transition area that is easily identified between the transparent cornea and the adjacent conjunctiva [5]. As the niche environment of limbal epithelial stem cells (LESC) [6], structural changes of palisades of Vogt (POV) located in the limbus may precede the early clinical manifestations of ocular surface diseases [7,8]. A better understanding of biological characteristics of the limbus will provide great insight into the pathogenesis of diseases, such as pterygium. In recent years, there has been increasing interest in limbal epithelial thickness changes in different quadrants of the limbus because limbal epithelial thickness changes were determined to cause abnormal corneal refraction [9] and indicate limbal damage, such as that which is associated with ultraviolet light or repeated exposure to chemical surfactants throughout life [10,11]. Therefore, the evaluation of limbal epithelial thickness has potential value for identifying early changes associated with ocular surface diseases, evaluating corneal damage and disease risk, and improving treatment methods. Previous studies have analyzed the relationship between the limbus and age [12]. However, changes associated with age are not consistent in different positions of the central cornea. Therefore, we aim to evaluate the utility of a new parameter that compares limbal epithelial with central corneal marginal epithelial thickness that may be used to assess how the limbus is affected by age and ocular surface diseases, such as pterygium.

Optical coherence tomography (OCT) has proved to be a useful non-invasive adjunctive tool for observing changes of limbal area, which may be directly affected by the limbal epithelial stem cell microenvironment. In this research, we compared limbal epithelial thickness values of different age groups of patients with pterygium and healthy controls via OCT imaging to assess the utility of an epithelial thickness of POV (POV-ET)/epithelial thickness of BM (BM-ET) ratio (POV-ET/BM-ET), as a new index for evaluating effects of pterygium and age on limbal epithelial thickness in different quadrants of the limbus.

## 2. Methods

### 2.1. Subjects

This is a cross-sectional study. Patients with pterygium were recruited from Peking University Third Hospital Eye Center from 2019 to 2020. Age-matched, healthy controls were also recruited. Inclusion criteria applied when selecting patients of the pterygium group were as follows: (1) diagnosis of pterygium; (2) without history of ocular trauma or surgery; and (3) without obvious arcus lipoides. The inclusion criterion for individuals of the healthy control group was no ocular pathologies. All clinical records of participants, including general ophthalmological examinations and OCT imaging findings, were collected. The study was approved by Peking University Third Hospital Medical Science Research Ethics Committee, and the implementation process adhered to the tenets of the Declaration of Helsinki.

### 2.2. OCT Imaging and Epithelial Thickness Measurement

An OCT system (Avanti; Optovue Inc., Fremont, CA, USA) with a corneal anterior module long adaptor lens was used in the present study. The system had a scan speed of 70,000 axial scans per second, and the resolution was 5 μm in tissue. Participants were asked to look at indicator lights in each direction to measure the status of cornea limbus and explore differences in the niche environment among the superior, nasal, inferior, and temporal regions using cross-line scan examination mode.

**Figure 1:**
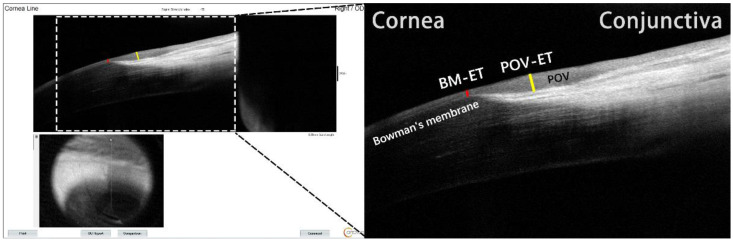
A diagram depicting the measurement of Bowman’s membrane epithelial thickness (BM-ET) and palisades of Vogt epithelial thickness (POV-ET). Red and yellow lines indicate BM-ET and POV-ET, respectively.

The corneal epithelium and the POV are typically assessed when evaluating the status of the ocular surface and human corneoscleral limbus. The former could be clearly observed in all subjects, while the normal structure of the latter could not be accurately detected, especially within the nasal quadrant of the limbus of patients with pterygium. BM-ET was used to indicate the state of the corneal epithelium and serve as a reference value reflective of any change in limbal position of corneal stem cells. POV-ET was used to reflect the niche environment of limbal epithelial stem cells and the current state of limbal epithelial stem cells. In this study, we manually measured the vertical distance from the end of BM to the corneal surface as BM-ET in all quadrants for all participants. POV-ET was defined as the maximum vertical distance of the POV to the corneal surface at the corneal-limbal junction. (Figure 1) Values of all quadrants of all groups were measured, except for the nasal quadrant of pterygium groups. Each measurement was repeated three times and averaged by the same examiner. POV-ET/BM-ET was calculated to evaluate the effect of aging and pterygium on the corneal limbus.

### 2.3. Statistical Analysis

Statistical analyses were conducted using IBM SPSS 26.0 and R 4.0.4 software. Continuous variables were presented as mean ± standard deviation (SD) and differences between groups were compared via two-tailed t-tests and one-way analysis of variance. Categorical data were described as ratios and assessed using the Chi-squared test. Relationships between variables were evaluated using Spearman’s rank correlation coefficient. Values of *p* < 0.05 were considered statistically significant.

## 3. Results

**Figure 2:**
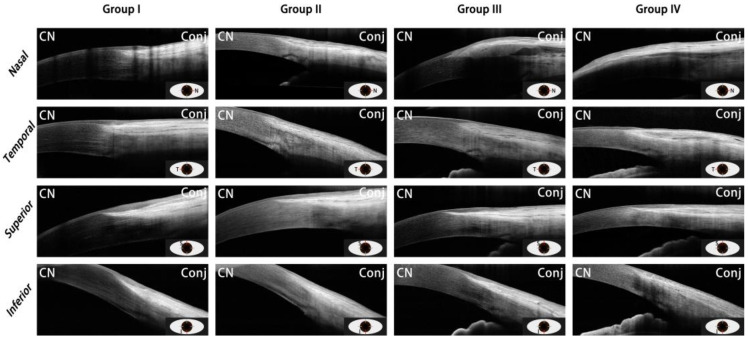
Representative OCT images of each quadrant of each of the four groups are shown. All images were of the right eye of subjects. The images of the following participants are shown: Group I, 22-year-old male healthy subject; Group II, 65-year-old female healthy subject; Group III, 36-year-old male pterygium patient; Group IV, 65-year-old male pterygium patient. CN, cornea; Conj, conjunctiva; OCT, optical coherence tomography.

In this study, a total of 35 eyes of 21 pterygium patients were enrolled in the case group and 45 eyes of 27 healthy volunteers were included in the healthy control group, according to the principle of age matching. All patients were divided into the following four groups based on age and disease status for further statistical analysis: (I) healthy volunteers aged < 60 years; (II) healthy volunteers aged ≥ 60 years; (III) pterygium patients aged < 60 years; (IV) pterygium patients aged ≥ 60 years. Table 1 includes demographic data of all subjects. No age or gender differences among groups were observed. Figure 2 shows the representative OCT images of four groups in each quadrant.

### 3.1. POV-ET/BM-ET

Table 2 shows POV-ET/BM-ET parameter values calculated. There were missing values regarding the nasal quadrant of pterygium patients (Group III and IV) because POV-ET could not be measured accurately. Among four quadrants of all groups, the superior quadrant had the highest POV-ET/BM-ET value, followed by the inferior quadrant. Nasal and temporal quadrant values were similar, and lower than those of superior and inferior quadrants. In addition, superior and inferior quadrant values exhibited a relatively large degree of variability, with greater standard deviations, versus other quadrants.

### 3.2. Within-Group POV-ET/BM-ET Value Correlations

**Figure 3:**
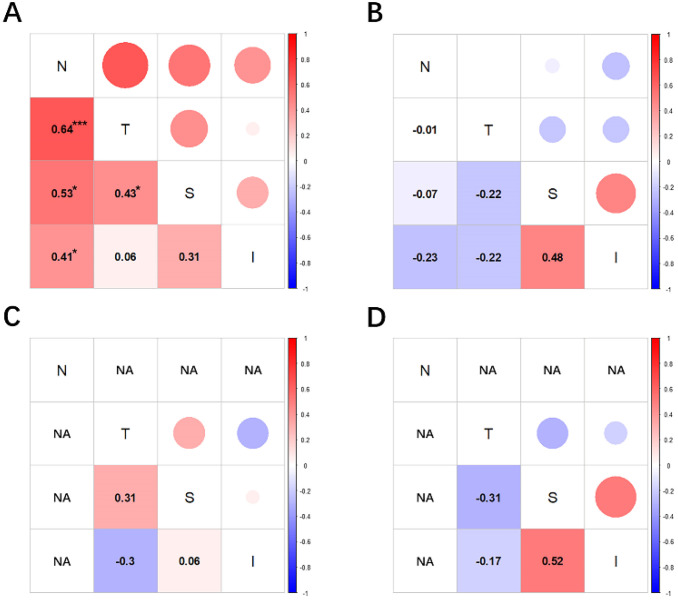
Spearman correlations among palisades of Vogt epithelial thickness/Bowman’s membrane epithelial thickness (POV-ET/BM-ET) values of quadrants of each group are shown. Red indicates a positive correlation, blue indicates a negative correlation, and increasingly dark colors indicate increasingly significant correlations. (**A**): Correlations between POV-ET/BM-ET and age among Group I, the only one of the four groups considered in which the correlation was observed. (**B**): Correlation between POV-ET/BM-ET values and age among Group II. (**C**): Correlation between POV-ET/BM-ET and age among Group III. (**D**): Correlation between the POV−ET/BM−ET and age among Group IV. N, nasal; T, temporal; S, superior; I, inferior; ***, *p* < 0.001; *, *p* < 0.05.

Figure 3 shows correlations between quadrants of different groups. Group I had the highest degree of intra-group association of POV-ET/BM-ET values, especially for the nasal side, with values that highly or moderately positively correlated with all quadrants. POV-ET/BM-ET values of the temporal side only moderately positively correlated with those of the nasal quadrant and did not correlate with those of the superior and inferior regions. Negative internal correlations between POV-ET/BM-ET values were observed among other groups; however, their Spearman’s coefficients were not determined to be statistically significant.

### 3.3. Between-Group Differences in POV-ET/BM-ET

**Figure 4:**
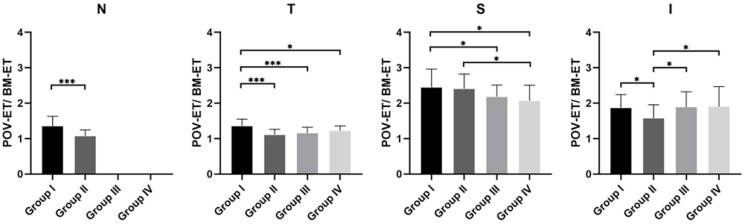
Between-group differences in palisades of Vogt epithelial thickness/Bowman’s membrane epithelial thickness (POV-ET/BM-ET) values in each quadrant. N. nasal; T, temporal; S, superior; I, inferior; ***, *p* < 0.001; *, *p* < 0.05.

Between-group comparisons between POV-ET/BM-ET values are shown in Figure 3. For the nasal quadrant, Group I values were significantly greater than those of Group II, with a mean difference of 0.28. For the temporal quadrant, POV-ET/BM-ET values of Group I were the greatest among all groups, while no between-group differences among Groups II, III, or IV were identified. In the superior quadrant, Group III and IV values were significantly lower than those of Group I. Further, Group IV values were lower than those of Group II. Notably, regarding the inferior quadrant, values of Group II were lower than all other groups considered. Finally, no between-group differences among the other three groups were identified. (Figure 4).

## 4. Discussion

OCT provides a more rapid and accurate means to explore the structure of the corneal limbus under pathological conditions than ex vivo histologic studies because it allows for the omission of the fixation and dehydration process [13]. In the current study, we evaluated the status of the corneal limbus of patients with pterygium via OCT and further explored potential effects of age. Our results indicate POV-ET/BM-ET values of all quadrants are correlated in young healthy subjects, a finding not observed among other groups. POV-ET/BM-ET values of patients who suffered from pterygium were reduced in the temporal and superior quadrant and elevated in the inferior quadrant. Aging was associated with reductions in nasal, temporal, and inferior quadrant values.

**Figure 5:**
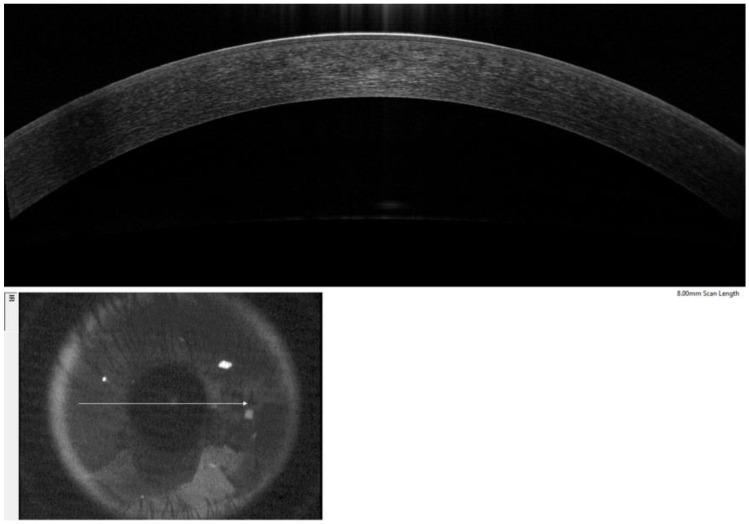
An optical coherence tomography image of the central cornea shows that the epithelial thickness of the central corneal epithelium is more uniform than that of the limbal epithelium.

In this study, POV-ET and BM-ET parameters were used to quantify the state of POV and adjacent central corneal structure, respectively. Therefore, POV-ET/BM-ET was useful as an index value indicative of atrophy of POV structure, relative to central corneal thinning (Figure 5). Decreased parameter values indicated that POV atrophy was greater than that of the central cornea. Our results showed POV-ET/BM-ET values varied based on quadrant type, indicating that the anatomic structure of the limbus of different quadrants may vary. This suggests that targeted prevention and treatment methods may be useful for treating patients with limbal injuries in different regions.

Results of the correlation analysis of POV-ET/ BM-ET among each group showed that internal homeostasis among quadrants of young healthy corneal limbus structures may occur, especially within nasal and temporal quadrants. This finding indicates that anatomic structures of the corneal limbus of young, healthy individuals might be highly similar. However, the balance was disturbed due pterygium and aging. One possible cause of the imbalance in homeostasis may be that vertical eyelid compression due to blinking causes more severe and frequent damage to corneal limbal stem cells in aged eyes or those with pterygium [14]. It has also been suggested that variation in the distribution of limbal stem cells at the limbus may occur. As a result, in superior and inferior quadrants, in particular, the POV has an increased number of limbal stem cells [15]. Increased numbers of limbal stem cells improve the capacity of the eye to repair its epithelium. As a result, damage accumulates with age at a steady rate; however, the degree to which damage is repaired varies among quadrants. Therefore, correlations between quadrants may be lost with age. Further, we speculate that supplementation of limbal stem cells may be benefit patients with limbal injury. Ghouali et al. [16] reported that dry eye diseases similarly affected the POV structure of all quadrants. However, in the current research, between-quadrant POV-ET/ BM-ET associations disappeared with age, implying that anatomical structures of limbal quadrants may differ. For example, the POV of superior and inferior quadrants have more limbal stem cells than the other quadrants considered; therefore, structural changes of quadrants may differ in response to similar damage. Therefore, different quadrants of the limbus tend to be more sensitive to certain risk factors of disease than others, providing new ideas for improving our understanding the pathogenesis of quadrant-specific diseases, such as pterygium.

With increasing age, POV-ET/BM-ET values of all quadrants decreased, except for those of the superior quadrant. This indicates that the exhaustion of limbus is greater than that of central cornea within the nasal, temporal, and inferior quadrants. Further, the reason for this exhaustion may be the same. These findings are similar to those of Lin et al. [17], except the previous report showed no age-specific difference in the thickness of central corneal epithelium, while the thickness of limbal epithelium decreased with age. The thinning of the superior-central corneal thinning with age may prevent POV-ET/BM-ET parameter decreases of the superior quadrant because both POV-ET and BM-ET decrease with age. Le et al. [18] reported that limbal epithelial thickness is associated with the status of POV structure and speculated that limbal epithelial thinning may result from limbal stem cell deficiency. Investigations into the normal limbus suggested that the diameter and density of cells in the limbus changed with advancing age, whereby the mean diameter increased as cell density decreased [19]. These results suggest that the proliferative potential of LESCs at the POV decreases with age, diminishing their capacity to maintain the integrity and stability of corneal epithelium. This may explain why POV-ET/BM-ET values of elderly healthy subjects were lower than those of young healthy subjects. Further indicating that the effect of age on the niche of LESCs is usually negative; therefore, good therapeutic results using stem cells are difficult to achieve in elderly patients with limbal stem cell deficiencies without restoring an appropriate stem cell niche [20].

Our results showed that even if pterygium only occurred on the nasal quadrant, an associated decrease in temporal POV-ET/BM-ET was also observed. Reid and Dushku [21] reported that ultraviolet damage of the nasal limbus via the temporal limbus causes mutations in epithelial stem cells, resulting in pterygium. Moreover, King-Smith et al. [22] proposed that nasal tear flow temporally carrying substances, such as cytokines, to the nasal limbus may be involved in the pathogenesis of pterygium. It is possible that when substances carried by tears reach the nasal limbus, some also remain in the POV of the temporal limbus, which is directly affected by ultraviolet exposure. This process may be involved in temporal epithelial thickening, which was identified in patients with pterygium and infrequently occurred in temporal pterygium.

In this study, only the superior limbus of healthy subjects and patients with pterygium significantly differed. Many studies have shown that tissue from the superior region may be used for conjunctival autograft in the treatment of pterygium [23,24]. Sakarya et al. [25] reported that superior corneal quadrant sensitivity did not change before or after superior conjunctival autograft. Further, two months after the operation, superior conjunctival quadrant sensitivity returned to a normal level. Zhao et al. [26] reported that tear film in patients with pterygium was unstable and that superior corneal sensitivity diminished insignificantly before and after pterygium excision. Therefore, we believe that the substances that cause pterygium also reach the POV of the superior limbus via tear flow. This may explain the fact that significant levels of superior limbal epithelium thinning occur due to pterygium [4].

Compared to the healthy subjects, patients with pterygium had significantly increased POV-ET/BM-ET values in the inferior quadrant. Levy et al. [27] performed epithelial thickness mapping in patients with pterygium. The results of the study indicated that that the inferior epithelium of patients with pterygium underwent a greater degree of thickening than healthy controls. Previous studies reported that this may be due to the thickening of the epithelium or the underlying thickening of subepithelial mucosal layers of the POV [28,29]. Pterygium is characterized by Bowman’s membrane dissolution accompanied by abundant active fibroblasts [30,31]. In the inferior quadrant of elder subjects, POV-ET/BM-ET values of patients with pterygium were increased relative to those of healthy subjects of the same age group. Therefore, we propose that the substances are produced by the dissolution of Bowman’s membrane, especially at the end of Bowman’s membrane adjacent to the POV and deposited at the POV of the inferior limbus by gravity or blinking. This may explain why the thickening of the POV of inferior limbus occurred in all groups of patients with pterygium.

In summary, our results revealed variable effects of aging and pterygium on quadrants of cornea via the measurement of POV-ET/BM-ET, indicating that substances produced due to ultraviolet exposure or pterygium in the cornea may affect other quadrants of the cornea via as tear flow or gravity. We also noted that the lower limit of POV-ET/BM-ET was close to 1.00, indicating that the limbal epithelial thickness (thicker than that of the central cornea) would eventually equal central corneal thickness. This may be due to the gradual disappearance of the niche under the epithelium to a point in which the only remaining layer of epithelial cells is within the limbus. We propose that a boundary may exist along an extension of Bowman’s membrane between the limbal stem cell niche and epithelial cells; however, this requires further histological evidence.

Limitations of this study were its small sample size and inexact age matching. Arcus lipoides may have slightly affected epithelial thickness of Bowman’s membrane values measured in this study. Further studies that aim to assess specific reasons for corneal limbus quadrant differences are needed. In addition, although many of our findings are statistically significant, the clinical significance of these differences is not clear. Lastly, the nasal quadrant of patients with pterygium was not evaluated because measuring the thickness of the nasal limbal epithelium of patients with pterygium is difficult.

## Figures and Tables

**Table 1:** Characteristics of all subjects.

Group	Eyes (*n*)	M/F	Age
Group I	29	10/19	28.66 ± 9.36
Group II	16	8/8	68.37 ± 7.20
Group III	22	6/16	52.09 ± 7.57
Group IV	13	6/7	68.08 ± 6.99

Group I, healthy volunteers aged < 60 years; Group II, healthy volunteers aged ≥ 60 years; Group III, pterygium patients aged < 60 years; Group IV, pterygium patients aged ≥ 60 years. M, Male; F, Female.

**Table 2:** Palisades of Vogt epithelial thickness (POV-ET)/Bowman’s membrane epithelial thickness (BM-ET) ratios of groups in each quadrant.

Quadrant	Group I	Group II	Group III	Group IV	Total
Nasal	1.36 ± 0.27	1.08 ± 0.17	-	-	-
Temporal	1.36 ± 0.19	1.11 ± 0.15	1.16 ± 0.16	1.22 ± 0.13	1.23 ± 0.19
Superior	2.45 ± 0.51	2.41 ± 0.41	2.18 ± 0.33	2.07 ± 0.43	2.31 ± 0.45
Inferior	1.87 ± 0.37	1.58 ± 0.37	1.90 ± 0.43	1.90 ± 0.56	1.83 ± 0.43

Group I, healthy volunteers aged < 60 years; Group II, healthy volunteers aged ≥ 60 years; Group III, pterygium patients aged < 60 years; Group IV, pterygium patients aged ≥ 60 years.

## Data Availability

Data presented in this study are available from the corresponding author upon request.
